# A Review of the Incidence Diagnosis and Treatment of Spontaneous Hemorrhage in Patients Treated with Direct Oral Anticoagulants

**DOI:** 10.3390/jcm9092984

**Published:** 2020-09-15

**Authors:** Kulothungan Gunasekaran, Venkat Rajasurya, Joe Devasahayam, Mandeep Singh Rahi, Arul Chandran, Kalaimani Elango, Goutham Talari

**Affiliations:** 1 Division of Pulmonary Diseases and Critical Care, Yale-New Haven Health Bridgeport Hospital, Bridgeport, CT 06610, USA; SUNNY.MANDEEP@gmail.com; 2 Division of Pulmonary Diseases and Critical Care, Multi-Care Pulmonary Specialists, Puyallup, WA 98372, USA; venkatk2711@gmail.com; 3 Division of Pulmonary Diseases and Critical Care, Avera Medical Group, Sioux Falls, SD 57105, USA; jvmd.smc@gmail.com; 4 Division of Pulmonary Diseases and Critical Care, Hurley Medical Center, Flint, MI 48532, USA; arulchandranmd@gmail.com; 5 Division of Cardiology, University of Nevada, Las Vegas, NV 89154, USA; kalaimani.elango@gmail.com; 6 Division of Hospital Medicine, Henry Ford Hospital, Detroit, MI 48202, USA; gouthamtalari@gmail.com

**Keywords:** anticoagulation, spontaneous hemorrhage, spontaneous bleeding, DOAC, apixaban, dabigatran, rivaroxaban

## Abstract

Anticoagulation carries a tremendous therapeutic advantage in reducing morbidity and mortality with venous thromboembolism and atrial fibrillation. For over six decades, traditional anticoagulants like low molecular weight heparin and vitamin K antagonists like warfarin have been used to achieve therapeutic anticoagulation. In the past decade, multiple new direct oral anticoagulants have emerged and been approved for clinical use. Since their introduction, direct oral anticoagulants have changed the landscape of anticoagulants. With increasing indications and use in various patients, they have become the mainstay of treatment in venous thromboembolic diseases. The safety profile of direct oral anticoagulants is better or at least similar to warfarin, but several recent reports are focusing on spontaneous hemorrhages with direct oral anticoagulants. This narrative review aims to summarize the incidence of spontaneous hemorrhage in patients treated with direct oral anticoagulants and also offers practical management strategies for clinicians when patients receiving direct oral anticoagulants present with bleeding complications.

## 1. Introduction

**Figure 1:**
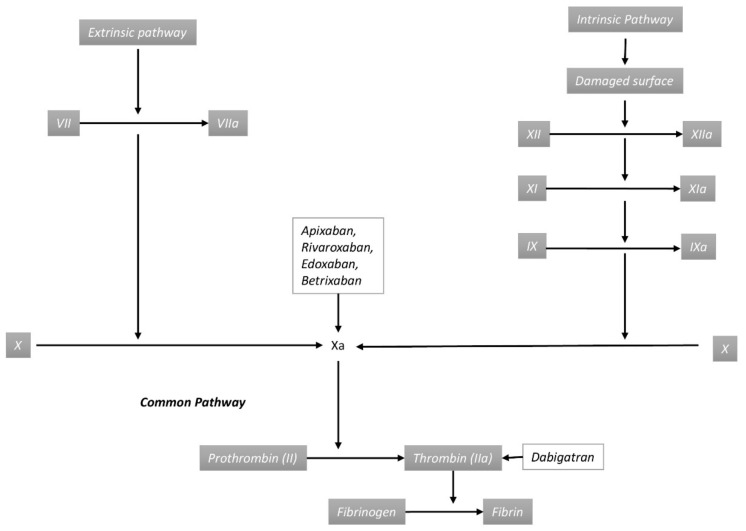
Mechanism of action of direct oral anticoagulants by inhibiting specific factors in the coagulation pathway.

Anticoagulation is a mainstay of treatment for thromboembolic disorders. It carries a tremendous therapeutic advantage in reducing morbidity and mortality associated with these disorders. Jay McLean first discovered heparin in 1916 [1]. For over six decades, traditional anticoagulants like unfractionated heparin (UFH) have been used for short-term anticoagulation. For patients who require long-term anticoagulation, low molecular weight heparin (LMWH), such as enoxaparin and vitamin K antagonists like warfarin, have been used to achieve therapeutic anticoagulation. Options for anticoagulation have been expanding steadily over the past decade with the approval of the first direct oral anticoagulant (DOAC) by the United States Food and Drug Administration (FDA) for stroke prevention in non-valvular atrial fibrillation, direct thrombin (factor IIa) inhibitor dabigatran in 2010, followed by direct factor Xa inhibitor rivaroxaban in 2011, apixaban in 2012, and edoxaban in 2015 [2]. This was followed by approval for venous thromboembolism (VTE) prophylaxis for patients undergoing hip and knee arthroplasty in the subsequent years. Most recently, a monoclonal antibody targeting factor XIa, Osocimab, was compared with apixaban and enoxaparin for venous thromboembolism (VTE) thromboprophylaxis in post-knee arthroplasty patients [3]. The selected characteristic features of approved direct oral anticoagulants (DOACs) are described in Table 1 and the mechanism of action is shown in Figure 1. Risk factors like malignancy, prolonged critical illness, immobilization, recent arthroplasty, and inherited thrombophilia increase the risk of VTE. Inherited thrombophilia are characterized by deficiencies of natural anticoagulants leading from specific genetic mutations, as described by Wypasek et al. [4]. Non-valvular atrial fibrillation and non-cancer associated VTE are the two most common and important indications for anticoagulation with DOACs.

No anticoagulant reduces thrombotic risk without simultaneously increasing the risk of bleeding to some degree. Bleeding is the most frequent complication of anticoagulant therapy, accountable for several hospitalizations and deaths. Major bleeding episodes, like gastrointestinal hemorrhage and intracerebral hemorrhage, carry significant mortality risk and are associated with prolonged hospitalizations and the need for invasive procedures. Clinically relevant non-major bleeding (CRNMB), on the other hand, carries significant morbidity, and the interruption of anticoagulation reduces the quality of life and patient compliance [5]. Spontaneous hemorrhage is bleeding or hematoma formation in a patient taking anticoagulation medication, which is atraumatic and non-intervention-related. Certain modifiable and non-modifiable risk factors are responsible for predisposing patients to bleeding.

The management of bleeding in individuals receiving a DOAC can be challenging because routine coagulation tests cannot be used to determine the degree of anticoagulation. Such treatment warrants close hemodynamic monitoring, the use of blood products, and specific reversal agents for DOACs. These reversal agents are expensive, maybe prothrombotic, and may not be readily available. In this review, we detail the epidemiology, incidence, pathogenesis, diagnosis, and management approach of spontaneous hemorrhage in patients receiving DOACs.

## 2. Indications for Modern Anticoagulants

The indications and use of DOACs have been expanding since they were first introduced. The American College of Chest Physicians (ACCP) guidelines on antithrombotic therapy for VTE disease recommend dabigatran, rivaroxaban, apixaban, or edoxaban as first-line agents for initial (first ten days, with and without initial heparin therapy) and long-term (first three months) anticoagulant therapy in non-cancer-associated DVT of the leg or PE [8].

The American College of Cardiology (ACC) recommends DOACs over vitamin K antagonists (VKA) in patients with non-valvular atrial fibrillation (AF) [9]. These recommendations stem from major randomized controlled trials favoring DOACs over standard VKA therapy. In a systematic review and meta-analysis for efficacy and safety outcomes done by Hulle et al. DOACs and VKA had similar efficacy in reducing recurrent VTE (RR 0.88; 95% CI 0.74–1.05, *P* = 0.46). However, the rate of major bleeding (RR 0.60; 95% CI 0.41–0.88, *P* = 0.03) and CRNMB (RR 0.76; 95% CI 0.58–0.99, *P* < 0.01) was lower for DOACs as compared to VKA [10]. In the United States, patients with atrial fibrillation and creatinine clearance (CrCl) of 15–50 mL/min, the recommended dose of rivaroxaban and edoxaban is reduced to 15 mg once daily with the evening meal and 30 mg once daily, respectively. In patients with severe chronic kidney disease (CrCl 15–30 mL/min), the recommended dose of dabigatran is reduced to 75 mg twice daily. In patients with end-stage renal disease (CrCl < 15 mL/min), all DOACs are not recommended except apixaban, with the dose reduced to 2.5 mg twice daily if either age is ≥80 years or bodyweight is ≤60 kg [9]. DOACs are contraindicated in patients taking enzyme-inducing antiepileptic drugs (e.g., phenytoin) and patients with HIV infection on protease inhibitor-based antiretroviral therapy.

ACCP 2016 guidelines recommend LMWH over VKA or DOACs for the first three months of anticoagulation in patients with VTE and cancer [8]. Recent trials published after the 2016 guidelines from ACCP may favor DOACs over LMWH [11,12,13]. For extended therapy in patients with VTE, ACCP guidelines recommend not changing the choice of anticoagulant after the first three months [8]. DOACs, mainly dabigatran, rivaroxaban, and apixaban, have been approved for VTE prophylaxis in patients after orthopedic surgery. Several randomized trials and meta-analyses compared efficacy and safety of DOACs with LMWH, aspirin, and fondaparinux in patients with total hip or knee arthroplasty. Indirect comparison of the various DOACs in a systematic review published in 2011 suggested that rivaroxaban may be more effective for DVT prophylaxis (RR 0.50; 95% CI, 0.37–0.68) when compared with dabigatran or apixaban but was associated with excess bleeding risk (RR 1.14; 95% CI, 0.80–1.64) [14,15]. The use of DOACs in patients with inherited thrombophilia and VTE has not been well documented in controlled trials. Gryn et al. described good outcomes in a young female with antithrombin deficiency and PE treated with rivaroxaban [16]. Wypasek et al. beautifully described two patients with protein S deficiency stemming from PROS-1 mutation treated with rivaroxaban. Both patients had progression of their thrombotic disease [17]. Indeed, genetic mutations in these inherited thrombophilia are distinct and more studies are required to describe the efficacy and adverse effects of DOACs use in this population. Although not robust, there is accumulating evidence from observational studies that DOACs may be effective in reducing thrombosis risk in heparin-induced thrombocytopenia (HIT). Two systematic reviews of observational data identified 100 patients with HIT who got treated with DOACs. From this observational data, the most significant experience was with rivaroxaban [18,19]. Further prospective studies are needed to determine the efficacy and safety of DOACs in such a patient population.

## 3. Epidemiology

Bleeding complications can be divided into either major or clinically relevant non-major bleeding (CRNMB) as reported in various systematic reviews and major randomized controlled trials (RCTs), observational studies, case series, and case reports. Based on recent systematic reviews (Table 2), the rate of fatal bleeding and major bleeding with DOAC use ranges from 0.06% to 0.30% and 1.1% to 4%, respectively. The rate of intracranial hemorrhage (ICH) ranges from 0.09% to 0.51%, major gastrointestinal (GI) bleeding from 0.35% to 2.09%, and CRNMB from 6.6% to 10.24% [10,20]. ICH is the most dreaded complication with DOAC use. A meta-analysis including more than 100,000 patients on DOACs found that dabigatran at a dose of 110 mg was safest of all and reduced the risk of ICH by 56% compared to rivaroxaban [21].

In one retrospective analysis by Khan et al. elderly (75+ years) patients taking DOACs were analyzed for bleeding complications. Five out of 142 patients had major bleeding (3 had gastrointestinal bleeding, 1 had a thalamic bleed, and 1 had hip hematoma after a fall). Twelve out of 142 patients had CRNMB events (4 had epistaxis, 3 had hematuria, 2 had vaginal bleeding, 1 had hematochezia, 1 had a subconjunctival hemorrhage, and 1 had easy bruising). Thirty-six percent of patients had moderate to severe renal failure, and all major bleeding episodes were associated with a decline in glomerular filtration rate compared with baseline [22]. Bleeding complications have been reported in patients taking lower doses of DOACs. In a study by Barra et al. out of 224 patients taking lower dose DOAC, 21 patients had gastrointestinal bleeding, 8 had epistaxis, 3 had hematochezia, 4 had intracranial hemorrhage, 4 had hematuria, 2 had hemoptysis, and 8 were unknown or other. Some patients had more than one bleeding event [23]. In a review of 50 patients taking DOACs, Franco et al. found that 20 had gastrointestinal bleeding, 17 had genitourinary bleeding (mainly hematuria), 6 had bleeding from the respiratory tract, 1 had an intramuscular bleed, and 5 had bleeding affecting the skin/subcutaneous tissue [24].

In a small retrospective analysis by Treder et al. the frequency of intraocular hemorrhage in patients taking apixaban or VKA (phenprocoumon) was compared. Retinal or vitreous hemorrhage occurred in 36% of patients taking apixaban and only 3.4% of those taking phenprocoumon [25]. In a retrospective review by Senger et al. from a total of 17 patients (7 on dabigatran and 1 on rivaroxaban), spontaneous intracranial hemorrhage occurred in 9 patients [26]. A comprehensive review by Godin et al. reported a higher risk of abnormal uterine bleeding with rivaroxaban as compared to VKA and no difference when apixaban was compared with VKA [27]. Kurogi et al. performed a cross-sectional survey in 2245 patients admitted across 621 hospitals in Japan to compare the outcomes of DOAC and warfarin-associated nontraumatic intracerebral hemorrhage. Patients with DOAC-associated ICH were less likely to suffer moderately or severely impaired consciousness (31.3% vs. 39.4%; *P* = 0.002) or require surgical intervention (5.3% vs. 9.9%; *P* = 0.024). The ICH associated with DOAC also had a lower mortality rate [28]. Caughey et al. analyzed the reporting of spontaneous adverse events (SAE) associated with apixaban use in Australia, Canada, and the United States. GI bleeding was the most commonly reported SAE (10% in Australia, 13% in Canada, and 8% in the USA), followed by cerebrovascular hemorrhage (7.2% in Australia, 2.6% in Canada, and 3% in the USA) [29].

Zaarour et al. reported a case of spontaneous spinal (cervicothoracic) subdural hematoma in a 58-year-old male who had a hip arthroplasty three weeks before under spinal anesthesia and was taking rivaroxaban 30 mg once daily for stroke prevention in atrial fibrillation [30]. Radcliff et al. reported a case of spontaneous lumbar epidural hematoma in a 53-year-old female who was treated with rivaroxaban for DVT prophylaxis after a routine revision of total knee arthroplasty under spinal anesthesia [31]. Atia R et al. reported two cases of spontaneous choroidal hemorrhage in patients taking rivaroxaban and dabigatran [32]. Hemorrhagic cardiac tamponade and spontaneous hemopericardium have been reported in patients receiving DOACs [33,34,35]. Jun et al. reported three cases of spontaneous vitreous hemorrhage in patients taking rivaroxaban for atrial fibrillation. All three patients were transitioning from warfarin to rivaroxaban when they experienced a spontaneous vitreous hemorrhage [36]. Hemoperitoneum from atraumatic splenic rupture and in the setting of acute cholecystitis in patients taking DOACs has been reported [37,38].

Gunasekaran et al. reported a case of spontaneous rectus sheath hematoma in a 68-year-old female taking apixaban for a provoked VTE after a recent knee replacement surgery. She required two units of packed red blood cell transfusion and an IVC filter placement as anticoagulation was discontinued [39,40]. Aktas et al. reported a case of spontaneous rectus sheath hematoma in a 71-year-old female with atrial fibrillation taking apixaban [41]. In both cases, there was no concomitant use of other antithrombotic or anticoagulant medications. Khan et al. reported a case of spontaneous retroperitoneal hemorrhage in an elderly female taking apixaban for atrial fibrillation, which required admission to the intensive care unit [42]. Table 3 below shows various bleeding complications from DOAC use, as reported in multiple RCTs and observational studies.

## 4. Bleeding

### 4.1. Bleeding Severity

Bleeding occurs in a spectrum ranging from CRNMBs or minor bleeding to significant or even life-threatening bleeding. The bleeding may be spontaneous, or it may be related to surgery. Hemoglobin concentration cannot initially be used to measure the severity of bleeding because the development of anemia will be delayed until fluid resuscitation or rebalancing from the body’s water, usually in the case of mild bleeding. Thus, clinical judgment is required in all cases of bleeding to determine the risk of the patient and whether the bleeding is resolving or worsening.

It is essential to have a standardized approach in defining and comparing major bleeds and CRNMBs when analyzing clinical data from randomized controlled trials and systematic reviews. These definitions can be extrapolated to clinical practice to streamline the management of patients experiencing bleeding and other adverse events from anticoagulation use. The International Society on Thrombosis and Hemostasis (ISTH) definition for major bleeding has been extensively used in major clinical trials involving DOACs [43]. The Thrombolysis in Myocardial Infarction (TIMI) criteria have been used in cardiovascular trials [44]. The Global Utilization of Streptokinase and Tissue Plasminogen Activator for Occluded Coronary Arteries (GUSTO) criteria for bleeding severity have also been used in various clinical trials, especially the early STEMI trials involving fibrinolytic therapy [45]; overall, it is less commonly used than the other two. Recently, the Bleeding Academic Research Consortium (BARC) has developed a highly standardized bleeding severity definition mainly for cardiovascular clinical trials and patients receiving anticoagulant therapy [46] (Table 4).

### 4.2. Pathogenesis of Direct Oral Anticoagulant-Associated Bleeding

Hemostasis is a balance between procoagulant and anticoagulant forces, and consists of mechanisms that maintain steady vascular blood flow. This is achieved through primary hemostasis, which forms the platelet plug, and secondary hemostasis that requires the formation of a fibrin clot through a chain of enzymatic reactions [47]. Anticoagulants interfere with the normal hemostatic process and lead to excessive bleeding and hematoma expansion after a disruption in the vessel wall integrity and alteration in vascular endothelium. This could happen from mechanical causes (trauma, tumor invasion, thrombosis, hypertension, invasive vascular procedures) or from an alteration in the endothelial cell barrier function (sepsis, hypoxia, ischemia, drugs like nonsteroidal anti-inflammatory drugs (NSAIDs), chemotherapeutic agents, infections, etc.,) [48]. Microbleeds are common in the brain and can also occur in other organs like the mucosal lining of the gastrointestinal tract. Cerebral microbleeds are small asymptomatic chronic brain hemorrhages caused by structural small vessel abnormalities and can be seen in hypertensive vasculopathy and cerebral amyloid angiopathy. Cerebral microbleeds have been associated with a higher incidence of intracranial hemorrhage [49]. Microbleeds are not uncommon in the mucosa of the gastrointestinal tract, and in patients taking DOACs, subclinical bleeding may present as clinically significant hemorrhage.

### 4.3. Risk Factors

The risk of bleeding with DOAC depends on several factors that can be broadly divided into those related to the anticoagulant used and those related to patient characteristics. Factors related to the anticoagulant used include the type of DOAC used and the dosage. Patient-related factors include older age, associated comorbidities like renal failure, liver disease, malignancies, thrombocytopenia, and concomitant use of other medications that can increase the risk of bleeding (Table 5). Overall, the risk of life-threatening bleeding is lower with DOACs when compared with warfarin. The risk of bleeding after anticoagulation initiation is highest during the first three to six months and regular follow-up is required [50]. In patients with a high risk of bleeding, the total risk of bleeding was 12.8% during the first three months and the annual rate was reduced to slightly over 6.5% after three months of initiation of anticoagulation treatment [51]. Elderly patients have multiple coexisting comorbidities and are on numerous medications with an increased risk for drug interactions. They also frequently have renal impairment, which would require dose adjustments according to the creatinine clearance rate. Overall, the elderly population with non-valvular AF has a higher risk of bleeding with DOACs compared with the younger people [52]. Patients with prior history of ICH and GI bleed are at increased risk for re-bleed. The risk of recurrent ICH in patients who have had ICH before is around 2.3% per year [53]. Patients with cirrhosis are at risk for bleeding secondary to coagulopathy and are also at risk for esophageal variceal bleed. With the advent of reversal agents, the risk of bleeding with DOAC is not much different than with warfarin [54]. Patients with chronic renal failure are at increased risk for bleeding from uremia and because of renal clearance, DOACs will need dosage adjustments. Dabigatran is most dependent and apixaban is least dependent on renal clearance [55]. However, despite these concerns, a meta-analysis of 45 trials reported that DOACs were more potent than warfarin in reducing the risk of stroke in CKD patients with AF without a significant increase in the risk of significant bleeding [56]. Because of the effects of diabetes on blood vessels, the risk of bleeding is slightly higher in this subgroup compared with the nondiabetics; however, there is not enough literature to estimate the risk of bleeding with DOACs in the subset. Malignancy increases the risk of bleeding because of increased vascularity, tumor invasion, and the release of inflammatory cytokines. A meta-analysis of 5000 patients with cancer and VTE found that the risk of bleeding was 4.9% in patients treated with DOACs [57]. A multicenter RCT performed in the United Kingdom found that at six months in patients with malignancy and VTE, the cumulative rate of major bleeding was 6% for rivaroxaban and CRNMB was 13%. In particular, GI and genitourinary cancers had a higher risk of bleeding with DOACs [11]. Thrombocytopenia and individuals with coagulation factor deficiencies are also at increased risk of bleeding. Patients with moderate to severe thrombocytopenia were excluded from three major DOAC trials. Sadowska et al. studied 62 patients with moderate to severe thrombocytopenia and AF receiving DOACs [58]. Similar rates of bleeding were seen compared with the normocytopenic control population. Interestingly, DOAC-induced thrombocytopenia has been documented in the literature [59]. Several trials compared the risk of bleeding with DOACs with concomitant antiplatelet medication use. APPRAISE-2 is a randomized, double-blind trial that compared apixaban 5 mg twice daily with placebo in addition to aspirin in patients with recent acute coronary syndrome [60]. However, this trial was terminated prematurely because of an increase in major bleeding events with apixaban (5 fatal bleeds and 12 episodes of ICH). The COMPASS trial compared rivaroxaban plus aspirin with rivaroxaban alone or aspirin alone in patients with stable atherosclerotic vascular disease [61]. Major bleeding events (mostly GI) were more common in patients in the rivaroxaban plus aspirin group (3.1%). There was no significant difference in intracranial or fatal bleeding between these groups. When compared with a combination of vitamin K antagonist and antiplatelet agents, the combination of DOAC and antiplatelet agents is associated with a similar risk of gastrointestinal bleeding and decreased risk of intracranial hemorrhage and major bleeding [62,63,64]. The incidence of ICH is increased in patients with hypertension, prior cerebrovascular accident, cerebral microbleeds as detected on magnetic resonance imaging (MRI), cerebral amyloid angiopathy, intracranial aneurysm, brain tumors, drug abuse, falls, intracranial infections, and septic emboli. Chronic obstructive pulmonary disease (COPD), cigarette smoking, and diabetes are risk factors for cerebral microbleeds. The use of DOAC increases the incidence of significant hemorrhage in patients with microbleeds [65]. The risk of GI bleed is increased in patients with GI tumors, alcohol use, NSAIDs use, smokers, presence of varices, peptic ulcer disease, gastritis, etc. DOACs increases the risk of bleeding in other gastrointestinal conditions associated with spontaneous hemorrhage like liver tumors, HELLP (hemolysis, elevated liver enzymes, low platelet count) syndrome, splenomegaly from hematologic malignancies, renal tumors, vasculitis, and spontaneous adrenal hemorrhage [7,66]. A meta-analysis of 43 RCTs found that GI bleeding risk was highest for rivaroxaban and dabigatran and lowest for apixaban [66].

### 4.4. Risk Reduction Strategies

Initiating and continuing anticoagulation is a multifaceted process that requires the consideration of several factors. The most important factor weighting is the risk of bleeding contrasted to the benefits of anticoagulation. Bleeding risk can be secondary to multiple factors and can also change with time, which makes it essential to revisit the goals of anticoagulation periodically [5,67]. A detailed discussion with the patient regarding their preference of route of therapy, upkeep of testing required with warfarin, cost barriers with the newer anticoagulants, as well as the patient’s underlying health conditions is vital in tailoring a plan for each patient. A patient’s adherence to medications should also be considered. Warfarin, as compared to DOACs, has less variability in its anticoagulant effects with a couple of missed doses [53,68]. A patient’s underlying conditions can sometimes dictate the selection of anticoagulants. Patients with mechanical heart valves, pregnant or breast-feeding patients, as well as patients with thrombotic risk secondary to the antiphospholipid syndrome are not candidates for DOACs [69,70]. Renal and hepatic dysfunction may also limit the use of DOACs. Medications that can interact with DOACs should be highlighted. A dose or medication change may be necessary to reduce the risk of bleeding [6]. Dose adjustments for some DOACs may be required when they are combined with medications that can inhibit CYP3A4 enzyme-like macrolides [71]. There are several bleeding scoring systems used to determine bleeding risk in patients receiving anticoagulants and that help guide shared decision-making and close monitoring in high-risk patients (Table 6). The HAS-BLED score has the best evidence for predicting bleeding risk. This score comprises measures of hypertension (systolic blood pressure >160 mm Hg), abnormal renal function, abnormal liver function, stroke history, bleeding history, labile INR, elderly (>65 years), medication use (antiplatelet drugs/NSAIDs), and concomitant alcohol use (≥8 drinks/week), with each scoring one point [72].

The indications for combination therapy with antiplatelet agents should also be thoroughly vetted and, where appropriate, antiplatelet agents should be discontinued to reduce the overall bleeding risk [73]. Patients must also be educated regarding the risk of bleeding with over the counter (OTC) use of NSAIDs, which must be limited if not avoided in favor of more selective Cox-2 inhibitors. Data suggest the use of a proton pump inhibitor (PPI) can reduce the risk of GI bleeding when used along with certain DOACs. A retrospective cohort study with 1.6 million patients found that the incidence of GI bleed was highest among patients on rivaroxaban, and the risk of upper GI bleed hospitalizations was significantly lower (RR 0.66) when concurrent PPI was used in patients receiving DOACs. The choice of DOACs should also vary depending on the patient’s risk factors for GI bleeding; for example, the rate of hospitalizations for severe GI bleeding was higher for rivaroxaban compared with other DOACs [61]. Other factors that should be included in the decision-making process, as well as an ongoing guide to continuing anticoagulation, is the fall risk of a patient and measures should be put in place to reduce the risk for patients required to be on DOACs [22,52]. As mentioned in the trials listed above, higher doses of DOACs have been associated with an increased risk of ICH and GI bleeding [6,7]. While anticoagulation invariably increases the risk of bleeding, it is also just as important to have patients on the appropriate anticoagulant as well as dose for their condition. A retrospective analysis of around 15,000 patients with AF on DOACs found that under dosing of apixaban had an increased risk of stroke with no significant decrease in their risk for bleeding [74].

## 5. Management

### 5.1. Diagnosis

The main challenge of the diagnosis of hemorrhage is the relative lack of symptoms until the hemorrhage is significant, especially when the bleeding is not external. Intracranial, intrathoracic, intra-abdominal bleeding could be life-threatening. Assessment of active bleeding, the location of the bleed, the type of agent that could have contributed to the bleeding, the half-life of the agent, the timing of the last dose, possible overdose (intentional or accidental), coexisting hepatic or renal diseases, concomitant use of medications that affect hemostasis, presence of bleeding diathesis, all would have a direct effect on morbidity [7]. The extent of the anticoagulation effect is vital in assessing the clinico-pathologic path of the bleed and planning the necessary intervention. Knowledge of the half-lives and the metabolism, including the excretion of the precipitating medication, would guide the response [26].

There is no pathognomonic clinical sign for internal bleeding. Hence, clinical assessment and judgment are vital in those patients. Chronic occult bleeding can pose greater morbidity and relatively less hemodynamic instability than an acute bleed and can remain relatively unnoticed in the early stages. In general, significant blood loss would result in headache, confusion, stiff neck, lightheadedness, dyspnea, or chest pain. Altered mental status in patients with DOACs, particularly with sudden onset, should prompt suspicion for intracranial bleed. Cullen’s sign—the presence of bruising or edema in the abdominal wall—could indicate an underlying intra-abdominal bleed [75]. Intra-thoracic bleed and pulmonary hemorrhage could present as hemothorax and hemoptysis, respectively [35].

Routine blood investigations would include a coagulation panel with platelet count, hemoglobin, hematocrit, prothrombin time, activated partial thromboplastin time, and thrombin time if dabigatran is the offending or suspected drug. If there are prolonged values in the coagulation panel, then treatment could be aimed to normalize them, expecting a favorable outcome [76]. Unfortunately, routine coagulation testing would not assess many of these agents’ effects. In severe hemorrhage in the setting of sepsis or trauma that could have triggered disseminated intravascular coagulation (DIC), measuring the levels of D-dimer and fibrinogen would be useful. Thromboelastography (TEG) is generally not recommended in this clinical situation because of a lack of data supporting its use [77].

Major bleeding results in low hemoglobin and hematocrit, though it could be many hours before the laboratory testing shows the effect. Hence, the presence of clinical features of shock is more sensitive than any laboratory testing.

Imaging remains the cornerstone of the diagnosis of bleeding. Computerized tomography (CT) is more sensitive than plain radiographs and is the gold standard [39]. Either non-contrast CT or an MRI can confirm intracranial bleed [26]. Gastrointestinal endoscopy (upper and lower) would help in the diagnosis of GI bleed [66]. Bleeding in the muscle causing compartmental syndrome can be diagnosed with the appropriate radiological investigation. However, if the possibility of a bleed is very high, then treatment should not be delayed in suspected cases just to make the diagnosis.

Close monitoring with a serial assessment of hemodynamics and blood parameters like hemoglobin would be essential in a relatively stable patient with possible internal bleeding.

### 5.2. Treatment

Severe bleeding would generally require management in the intensive care unit. It would be reasonable to admit hemodynamically stable patients with mild to moderate bleeding to the floor. Immediate discontinuation of the offending medication is essential for all patients, but the consequence of discontinuation of such agents due to thromboembolic events needs to be considered [78]. The half-lives of these DOACs are short; hence, there could be thrombotic events when withholding the agents even for a short period [40]. Therefore, in minimal bleeding, it may not be required to hold the anticoagulant at all. Most of such patients may not need a rapid reversal of the DOAC drug effect [79].

The concurrent use of another anticoagulant or an antiplatelet agent would increase the risk of a bleed. The use of nonsteroidal anti-inflammatory drugs (NSAIDs), norepinephrine, or serotonin reuptake inhibitors (SNRI or SSRI) also increases bleeding risk [80]. Blood transfusion should be promptly started after intravenous fluids to correct the shock and establish hemodynamic stability. Patients with shock and hemodynamic instability and those with intracranial hemorrhage and depressed consciousness would need mechanical ventilator support. The options of reversal agents and antidotes should be immediately explored if the contribution DOAC is known [26,78,79]. These reversal agents will be discussed in a separate section below.

The supportive and surgical management that is specific for the particular bleeding location is beyond the scope of this article and will not be discussed here.

### 5.3. Reversal Agents

DOACs do not act by the same pathway as warfarin; hence the administration of agents like vitamin K is not useful in the reversal of DOAC. The reversal agents are specific to the offending DOACs. If not available, then non-specific reversal agents can be used.

#### 5.3.1. Drug-Specific Reversal Agents

Specific reversal agents are available for some of the DOACs. Idarucizumab is an anti-dabigatran monoclonal antibody that assists in the reversal of dabigatran in emergencies. It is recommended only if the offending agent is known to be dabigatran and whose thrombin time is prolonged [79,81]. Andexanet alfa is a specific intravenous antidote for the reversal of factor Xa inhibitors. A recent RCT compared andexanet alfa and placebo among healthy volunteers after the therapeutic administration of rivaroxaban or edoxaban [79,82] and showed a quick and effective reversal of anticoagulation by andexanet alfa compared with the placebo. There were no reported adverse events and it was well tolerated.

#### 5.3.2. Non-Specific Reversal Agents

Non-specific reversal agents include antifibrinolytics (tranexamic acid, epsilon- aminocaproic acid), prothrombin complex concentrates (PCC), and desmopressin. PCC (non-activated) and activated PCC have plasma-derived clotting factors. PCC comes as a four-factor or a three-factor combination. These agents could be used in the treatment of factor Xa inhibitors like rivaroxaban, betrixaban, edoxaban, and apixaban [6,79]. If a three-factor PCC is used, then FFP could be added as the PCC has very little of factor VII. It has to be noted that andexanet alfa and PCC could be prothrombotic; hence, careful monitoring is essential. Clinicians should be aware of the fact that the Xa inhibitors are highly protein-bound; therefore, hemodialysis would not be beneficial in the removal of the drugs [83]. Transfusion of RBCs would need to correct shock from hemorrhage and replenish the blood loss. Platelets are not useful in the reversal of DOAC in patients with a normal platelet count but can be used in patients with dangerous thrombocytopenia. FFP could be used in severe life-threatening hemorrhage and as a part of massive transfusion protocols along with blood transfusion [6,79,84].

#### 5.3.3. Future Antidotes

A variant of factor Xa, called “FXa(116L),” has shown promising results in mouse models by restoring hemostasis by reversing the effects of factor Xa as well as direct thrombin inhibitors [85]. In addition, PER977 (arapazine/chiraparantag) is a novel drug currently being studied for reversal of the effect of factor Xa inhibitors [86]. There have been theories that PER977 does not reverse the anticoagulant effect but only reduces the bleeding [87]. Further investigations would provide more promising options for counteracting the effects of DOACs in the event of life-threatening bleeding. Table 7 describes the management of bleeding in patients receiving DOACs.

### 5.4. Restitution of Anticoagulation

After the successful arrest of bleeding and reversal of the anticoagulant effect of the DOAC, the complicated situation would be restarting these anticoagulants. Generally, the risk of bleeding versus the risk of thrombosis due to the original condition that prompted the use of DOACs in the patients should be weighed. But for very few people whose risk of bleeding might outweigh the benefit, most would require their DOAC to be recommenced for avoiding life-threatening thrombotic complications. Failure to resume anticoagulants could cause harmful effects [67]. However, in patients with a very low risk of thromboembolism, it would be reasonable not to restart the DOAC. The decision regarding restarting them would be based on the merits and downsides of the individual clinical situation.

In general, there is no definite optimum time frame that has been studied or recommended, especially for DOAC. For intracranial hemorrhage, the American Heart Association/American Stroke Association recommends withholding anticoagulant for at least four weeks [78]. However, it would be reasonable to restart in 7–14 days in gastrointestinal bleed. Apixaban may be the preferred option among the DOAC [67]. However, in the situation of massive GI or intrathoracic bleed, more prolonged withholding of anticoagulation would be needed, especially if the risk of thromboembolism is relatively low.

## 6. Summary

With an increase in the number of patients receiving anticoagulation therapy, physicians should be aware of these rare spontaneous bleeding complications, particularly in high-risk patients, which will enable the prompt recognition and management of these conditions. All these newly approved reversal agents and others in late-phase development specifically target DOACs. If these agents are not available, then cautious use of PCC may help in life-threatening bleeding.

## Figures and Tables

**Table 1:** Characteristics of direct oral anticoagulants [6,7].

Drugs	Dabigatran	Apixaban	Betrixaban	Edoxaban	Rivaroxaban
Mechanism of action	Direct IIa (Thrombin) Inhibitor	Factor Xa Inhibitor	Factor Xa Inhibitor	Factor Xa Inhibitor	Factor Xa Inhibitor
Onset of action	Within 30 min	~30 min	Within 30 min	Within 30 min	Within 30 min
Duration of action (h)	24–36	At least 24	At least 24	24	24
Baseline elimination half-life in hours	12–17	9–14	19–27	10–14	5–9 (young)/11–13 (elderly)
Dosage					
Non-valvular AF	150 mg twice daily	5 mg twice daily **		60 mg once daily	20 mg once daily with the evening meal
VTE treatment	Parenteral anticoagulation for 5–10 days; then dabigatran 150 mg twice daily	10 mg twice daily for one week, then 5 mg twice daily		Parenteral anticoagulation for 5–10 days; then edoxaban 60 mg once daily	15 mg twice daily with food for three weeks; then 20 mg once daily with food
VTE prophylaxis	110 mg for the first day, then 220 mg once daily	2.5 mg twice daily	160 mg on the first day, followed by 80 mg once daily, with food		10 mg once daily, with or without food
Best laboratory measurement	dTT, ECT	Anti-Xa	Anti-Xa	Anti-Xa	Anti-Xa

** Apixaban dose is reduced to 2.5 mg twice daily if two out of three criteria are met (serum creatinine is ≥1.5 mg/dL, age is ≥80 years, or bodyweight is ≤60 kg).

**Table 2:** Rate of bleeding events in two systematic reviews of several major phase-3 randomized controlled trials involving direct oral anticoagulants vs. vitamin K antagonist [10,20].

Author (Year of Publication)	Study Inclusion	Fatal Bleeding	Major Bleeding	ICH	Major GI Bleeding	CRNMB
Van Der Hulle et al. (2014)	Five randomized controlled trials (2 evaluating rivaroxaban; 1, dabigatran; 1, apixaban; and 1, edoxaban)	0.06% vs. 0.17% N_d_ = 12,197 N_k_ = 12,193	1.1% vs. 1.7% N_d_ = 12,197 N_k_ = 12,193	0.09% vs. 0.25% N_d_ = 12,197 N_k_ = 12,193	0.35% vs. 0.53% N_d_ = 12,197 N_k_ = 12,193	6.6% vs. 8.4% N_d_ = 12,197 N_k_ = 12,193
Chai-Adisaksopha et al. (2014)	Twelve randomized controlled trials (4 evaluating dabigatran; 4, rivaroxaban; 2, apixaban; and 2, edoxaban)	0.30% vs. 0.52% N_d_ = 57,850 N_k_ = 44,757	4% vs. 4.64% N_d_ = 57,850 N_k_ = 44,757	0.51% vs. 1.08% N_d_ = 57,850 N_k_ = 44,757	2.09% vs. 1.70% N_d_ = 53,753 N_k_ = 40,650	10.24% vs. 11.05% N_d_ = 45,774 N_k_ = 38,750

**Table 3:** Bleeding complications from direct oral anticoagulant use [10,20,22,23,24,25,26,27,30,31,32,33,34,35,37,38,39,42].

BLEEDING COMPLICATIONS	
**MAJOR BLEEDING**	Intracranial bleeding (subarachnoid hemorrhage, epidural hemorrhage, subdural hemorrhage, and intraparenchymal hemorrhage)
	Intraspinal hemorrhage
	Intraocular hemorrhage (retinal or vitreous hemorrhage)
	Hemorrhagic cardiac tamponade/hemopericardium
	Retroperitoneal hemorrhage
	Gastrointestinal hemorrhage
	Joint hematoma, traumatic or non-traumatic
	Hemoperitoneum, atraumatic splenic rupture
**CLINICALLY RELEVANT NON-MAJOR BLEEDING (CRNMB)**	Genitourinary–Hematuria, vaginal bleeding, abnormal uterine bleeding
	Respiratory tract–hemoptysis, gingival bleeding, epistaxis
	Intramuscular–Rectus sheath hematoma
	Skin/subcutaneous–Bruising

**Table 4:** Bleeding Academic Research Consortium (BARC) standardized definitions developed mainly for cardiovascular trials.

**BARC Definitions**		
Type 0	**No bleeding**	
Type 1	Bleeding that is not actionable and does not cause the patient to seek unscheduled intervention.	
Type 2	Any overt, actionable sign of hemorrhage requiring non-surgical medical intervention by a healthcare professional.	
Type 3	a	Overt bleeding plus hemoglobin drop of 3 to <5 g/dL (provided hemoglobin drop is related to bleed)
	b	Overt bleeding plus hemoglobin drop ≥5 g/dL (provided hemoglobin drop is related to bleed) Cardiac tamponade Bleeding requiring surgical intervention for control or intravenous vasoactive agents
	c	Intracranial hemorrhage confirmed by autopsy or imaging or lumbar puncture Intraocular bleed compromising vision
Type 4	CABG-related or perioperative intracranial bleeding within 48 h	
Type 5	a	Probable fatal bleeding
	b	Definite fatal bleeding

**Table 5:** Risk factors for spontaneous hemorrhage in patients receiving direct oral anticoagulants [7,48,49,62,66].

Patient-Related Risk Factors
Advanced age Low body mass Smoking Associated comorbidities like hypertension, chronic obstructive pulmonary disease, diabetes mellitus, renal failure, liver disease Previous gastrointestinal or intracranial bleeding Malignancies—tumor invasion Hematologic disorders Collagen vascular disorders Thrombocytopenia Concomitant use of other medications including steroids, nonsteroidal anti-inflammatory drugs, aspirin or clopidogrel

**Table 6:** Common bleeding scores used in patients receiving anticoagulants [7].

COMMON BLEEDING SCORES
HAS-BLED score
HEMORR_2_HAGES score
ATRIA score
ORBIT-AF score
ABC bleeding score

**Table 7:** Management of bleeding in patients receiving direct oral anticoagulants [6,82,83,85,86,87].

**General Measures**	Confirm DOAC intake history, the timing of the last dose, check for concomitant medicine, particularly antiplatelet drugs, assess for hemodynamic compromise, check the renal function, and oral activated charcoal (if the last dose within prior two hours)	
**Minor Bleeding**	Stop therapy, local hemostatic measures, supportive care, and monitoring	
**Moderate Bleeding**	All of the above and fluid resuscitation, blood transfusion, consider fresh frozen plasma transfusion, and consider hemodialysis for dabigatran	
**Major Bleeding**	All of the above; consider massive transfusion protocol with packed red blood cells, platelets, fresh frozen plasma, and other procedures/surgeries to achieve hemostasis	
	Specific antidotes	Dabigatran–Idarazcizumab
		Xa inhibitors (Apixaban, Rivaroxaban, Edoxaban)–Andexant alfa
	Non-specific reversal agents: 4 Prothrombin complex concentrates (PCC) [Factors II, VII, IX, and X], Tranexamic acid, epsilon- aminocaproic acid, Desmopressin	
**Future antidotes**	FXa(116L) for both factor Xa as well as direct thrombin inhibitors	
	PER977 (Arapazine/Chiraparantag) for factor Xa inhibitors	

## Abbreviations

AF	Atrial fibrillation
AF	Atrial Fibrillation
Anti-Xa	drug-specific assays
aPTT	Activated partial thromboplastin time
COPD	Chronic Obstructive Pulmonary Disease
CRNMB	Clinically relevant non-major bleeding
CYP	Cytochromeombin time
DOACs	Direct Oral Anticoagulants
ECT	Ecarin clotting time
GI	Gastrointestinal
GI	Gastrointestinal
ICH	Intracranial hemorrhage
ICH	Intracranial Hemorrhage
LMWH	Low Molecular Weight Heparin
Nd	The total number of patients in the DOACs group
Nk	the total number of patients in the vitamin K antagonist group
NSAIDs	Nonsteroidal Anti-inflammatory Drugs
PT	Prothrombin time
UFH	Unfractionated Heparin
VKA	Vitamin K Antagonist
VTE	Venous Thromboembolism
